# Modulation of gut microbiota and short-chain fatty acids by probiotics attenuates inflammation in endometriosis

**DOI:** 10.3389/fmicb.2025.1713258

**Published:** 2026-02-02

**Authors:** Xiaoli Dong, Fang Xie, Ping Li

**Affiliations:** Reproductive Medicine Center, Longyan First Affiliated Hospital of Fujian Medical University, Longyan, Fujian, China

**Keywords:** 16S rDNA, endometriosis, gut microbiota, inflammation, metabolomics, probiotics, short-chain fatty acids

## Abstract

**Introduction:**

This study investigated whether probiotics alleviate Endometriosis (EMs)-related inflammation by modulating the gut microbiota and short-chain fatty acids (SCFAs).

**Methods:**

An endometriosis model was established in SD rats, which were randomly divided into a normal diet group (NCD) and a probiotic group (NCD_Pro), with four rats per group. After a 4-week dietary intervention, serum and fecal samples were collected. Tumor Necrosis Factor (TNF)-α and Interleukin (IL)-6 levels were measured by ELISA, gut microbiota composition was analyzed via 16S rRNA sequencing, and fecal levels of nine SCFAs were quantified using GC–MS.

**Results:**

Probiotic supplementation significantly reduced serum levels of TNF-α and IL-6 (*P* < 0.05), but did not significantly affect body weight, body length, or lesion volume. Beta diversity analysis revealed significant structural differences in gut microbiota between the two groups (*P* < 0.05), while alpha diversity showed no significant difference. At the phylum level, probiotic intervention decreased the relative abundance of Firmicutes and increased that of Bacteroidota and Proteobacteria. At the family level, certain bacterial families showed opposite abundance patterns between the two groups. At the genus level, Bifidobacterium and Lactobacillus were significantly enriched in the probiotic group. Microbial co-occurrence network analysis indicated increased node number and connectivity along with enhanced network stability in the probiotic group. SCFA profiling showed decreased levels of butyric acid (BA) and caproic acid (CA), and a significant increase in isocaproic acid (4-MVA) in the probiotic group. Correlation analysis revealed a significant negative association between specific differential microbiota and 4-MVA (*r* < −0.6, *P* < 0.01).

**Conclusion:**

Probiotic intervention alleviates systemic inflammation in endometriosis by reshaping the gut microbiota structure, enhancing microbial network stability, and modulating the SCFA metabolism. Our findings underscore the role of the gut microbiota-metabolism-immunity axis in EMs pathophysiology and point to 4-MVA as a hypothesis-generating candidate metabolite that requires further validation.

## Introduction

Endometriosis (EMs) is an inflammatory estrogen-dependent disease that affects women of childbearing age, characterized histologically by the presence of endometrial-like tissue outside the uterus, such as in the ovaries, fallopian tubes, intestines, and bladder (Jiang et al., 2021; Qin et al., 2022). It not only manifests as gynecological symptoms such as dysmenorrhea and infertility, but also up to 90% of patients with endometriosis experience gastrointestinal symptoms, such as bloating, nausea, and constipation (Xholli et al., 2023).

So far, the exact cause of endometriosis remains not fully understood, and the search for the best treatment is still ongoing (Yalçin Bahat et al., 2022). Some researchers have found that there is a bidirectional relationship between the human gut microbiota and the development of endometriosis, which also exhibits characteristics of invasiveness and recurrence similar to those of malignant tumors (Kobayashi, 2023; Talwar et al., 2022). In recent years, an increasing amount of evidence has also supported the link between endometriosis and dynamic changes in the gut microbiome (Weber et al., 2023). The gut microbiota plays a crucial role in maintaining mucosal integrity, preventing bacterial translocation, and regulating immune status (Pai et al., 2023), dysbiosis of the gut microbiota can affect immune responses and estrogen metabolism, and may play a key role in the pathogenesis and progression of endometriosis (Pai et al., 2023). Studies have shown that there are alterations in the gut microbiota of patients with endometriosis, including reduced diversity and imbalances in microbial composition. Compared with healthy women, patients with endometriosis usually have lower gut microbiota diversity. Alterations in the gut microbiota may lead to chronic inflammation, thereby promoting the progression of endometriosis (Xholli et al., 2023).

In addition, there is a close relationship between the gut microbiota and short-chain fatty acids (SCFAs). SCFAs, also known as volatile fatty acids, are primarily dependent on the composition of the gut microbiota, digestion time, host microbial metabolic flux, and the fiber content in the host diet. They mainly include acetate, propionate, and butyrate (Mukhopadhya and Louis, 2025). The gut microbiome produces SCFAs through the fermentation of dietary fiber, and these SCFAs collectively help maintain gut health and overall metabolic balance in the body (Di Costanzo et al., 2021; Tao and Wang, 2025). SCFAs may influence endometriosis through various pathways. First, SCFAs have anti-inflammatory effects, particularly butyrate, which can inhibit the production of pro-inflammatory cytokines and reduce inflammatory responses (Sam et al., 2021), this can help alleviate the symptoms of endometriosis. Second, SCFAs can also regulate the function of immune cells, including T cells, B cells, and macrophages. They can promote the production of regulatory T cells (Tregs), which play an important role in maintaining immune tolerance and suppressing autoimmune reactions (Smith et al., 2013). Third, supplementing with SCFAs can improve the composition of the gut microbiota, increasing the number of beneficial bacteria and reducing the number of harmful bacteria, thereby maintaining gut microbiota balance (Mukhopadhya and Louis, 2025). Probiotic diets have a significant impact on the gut microbiota, as they can alter its composition by increasing the number of beneficial bacteria and reducing the number of harmful bacteria. They can also enhance the gut microbiota's ability to ferment dietary fiber, thereby increasing the production of short-chain fatty acids and positively affecting the host's health (Bañares et al., 2025; Wang et al., 2024).

In summary, modulating the gut microbiota and its metabolite SCFAs may influence the occurrence, progression, and even treatment of endometriosis. However, due to the lack of clinical evidence, a substantial number of animal experiments are still needed to support this idea. Therefore, this study utilized an experimental rat model of endometriosis to investigate the impact of probiotic dietary intervention on gut microbiota structure and to identify different short-chain fatty acids. In addition, we will conduct correlation analyses between different microbial species and metabolites, measure the levels of TNF-α and IL-6 in serum using enzyme-linked immunosorbent assay (ELISA), and analyze the correlation between inflammatory markers and different microbial species. Although previous studies have indicated that the gut microbiota plays a positive role in regulating the progression of endometriosis, the specific effects of probiotics on this disease remain unclear. Thus, the primary objective of this study is to evaluate whether probiotics can alleviate the inflammatory response of endometriosis by improving the gut microbiota and its metabolism.

## Materials and methods

### Endometriosis rat model

All experimental protocols were approved by the Institutional Animal Care and Use Committee (IACUC) and followed the Guide (GUIDE) for the Care and Use of Laboratory Animals. In this experiment, 6-week-old female SD rats [purchased from Beijing Hua Fukang Biotechnology Co., Ltd., license number SCXK (Beijing) 2019-0008] were used. The rats were housed with standard food in an environment maintained at 20–26°C and 40%−70% humidity. After 1 week of acclimatization (week 1), a rat model of endometriosis was established. The donor rats were euthanized by decapitation, and the uterus was removed. The Y-shaped midline of the uterus was cut open, and the endometrium from both sides was carefully peeled off and preserved for transplantation.

**Figure 1:**
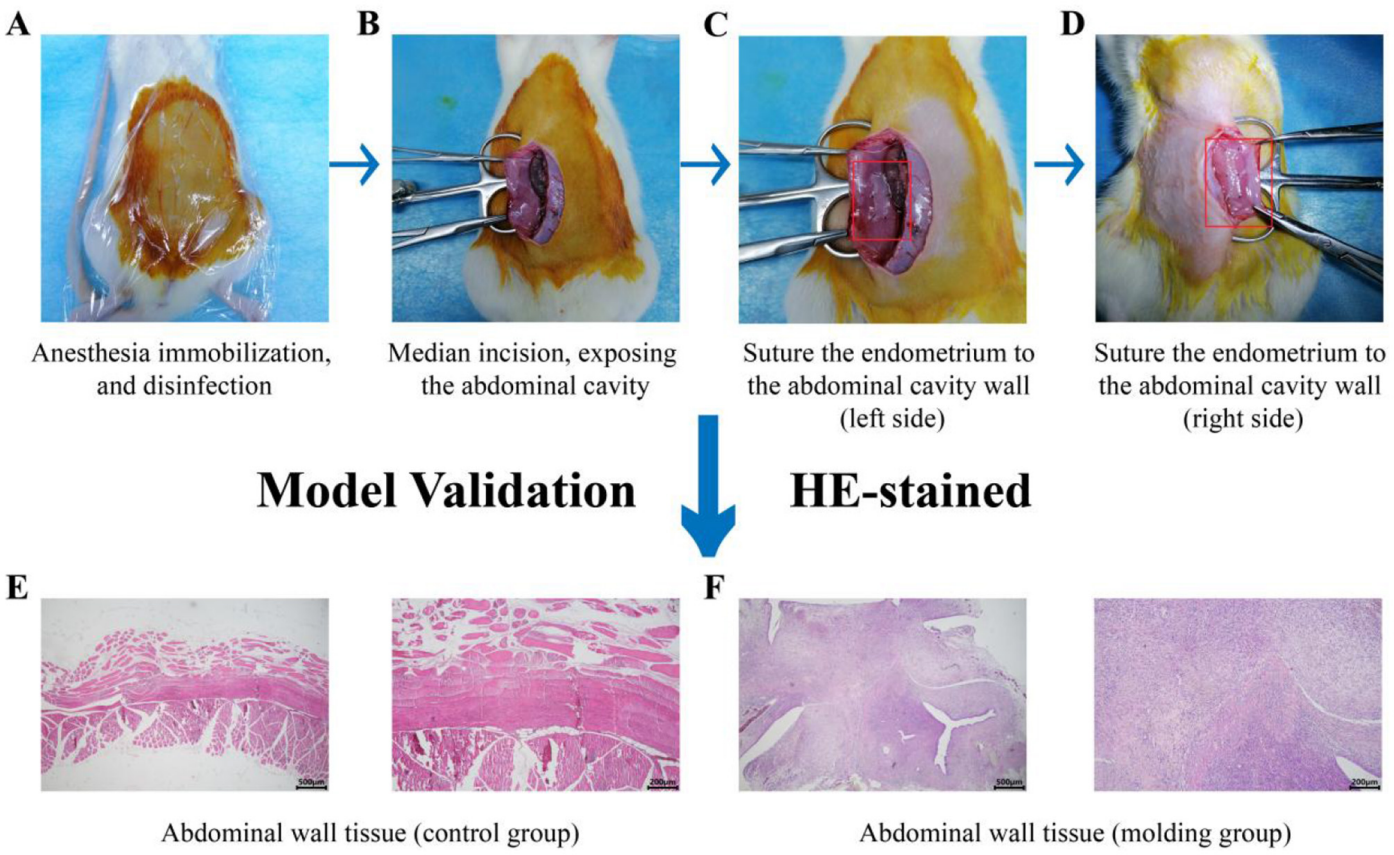
Modeling flowchart: **(A)** Anesthesia and immobilization, abdominal shaving and disinfection; **(B)** Midline incision, exposure of the abdominal cavity; **(C, D)** Suture the endometrium to the abdominal cavity wall (bilaterally); **(E)** HE-stained abdominal wall tissue (control group); and **(F)** HE-stained abdominal wall tissue (molding group).

The recipient rats were anesthetized, their abdomen shaved and prepared for surgery. The surgical site was disinfected with iodine, and a midline incision was made to expose the abdominal wall. The donor endometrial tissue was sutured to the abdominal wall of the recipient rats. The procedure was performed bilaterally, and after suturing, the abdominal cavity was closed layer by layer (Figures 1A–D).

Animal inclusion and exclusion criteria: all female SD rats that successfully underwent the endometriosis modeling surgery and recovered without complications during the 1-month post-operative acclimatization period were included in the study. Rats were excluded from the final analysis based on the following pre-established criteria: (1) death during or within 24 h after the modeling surgery; (2) presence of severe post-surgical complications (e.g., infection, self-mutilation of the wound, or failure to thrive).

Model validation: a randomly selected model rats was dissected to visualize vesicle formation. Lesion tissue was subjected to Hematoxylin and Eosin (HE) staining. Results revealed that the cavity surface structure in the model group resembled that of *in situ* endometrium, accompanied by secretions and inflammatory cell infiltration. The ectopic lesions were typical, confirming successful model establishment (Figures 1E, F).

**Figure 2:**
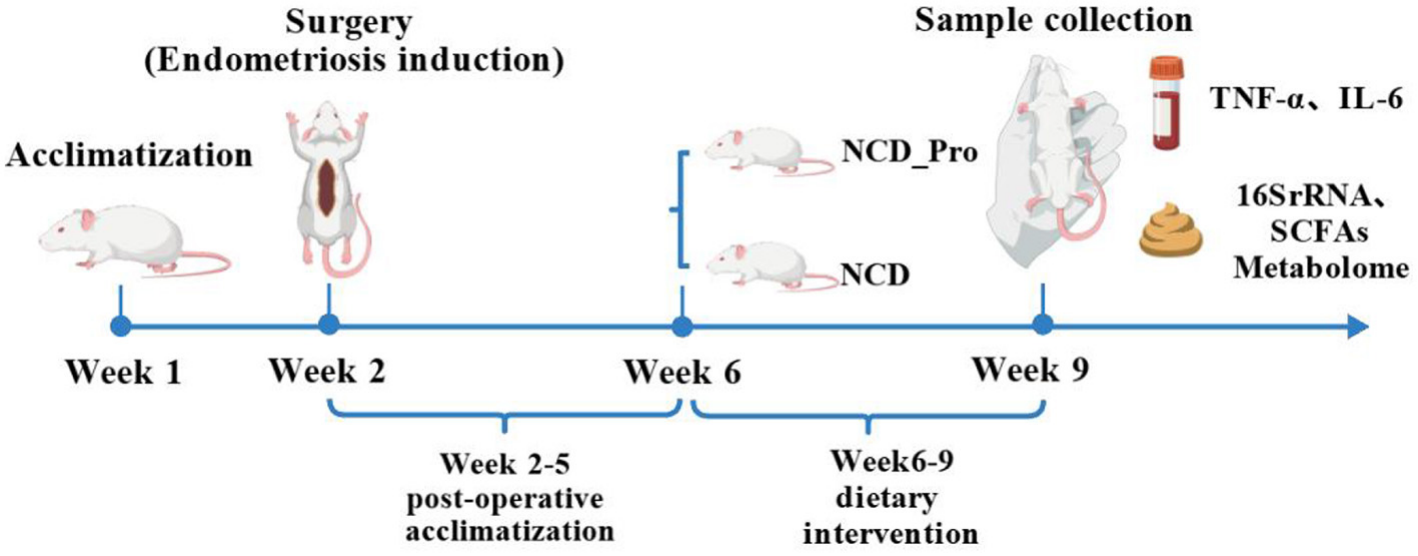
Schematic representation of the experimental design with different diet treatments in SD rats.

After modeling, the rats were acclimatized for 4 weeks (weeks 2–5), then randomly divided into two groups: a regular diet group (NCD) and a probiotic group (NCD_Pro), with four rats per group. The regular diet group was fed standard food, while the probiotic diet group received a probiotic supplement (0.75 mg/1 mL per rat) along with regular food. The laboratory provides standard feed composed of 60% carbohydrates (sucrose, cellulose, etc.), 20% protein (primarily casein), and 20% fat (soybean oil, lard, etc.). All feed undergoes irradiation sterilization and is stored in a refrigerator at 2–8 °C. It should be removed in advance when needed. The probiotic employed is a pet nutritional supplement designed for small animals, including hamsters; its main ingredients are *Bacillus subtilis* (≥4.0^*^10 cfu/g), *Lactobacillus acidophilus* (≥1.0^*^10 cfu/g), glucose, and vitamins [Manufacturer: Sichuan Feitian Animal Pharmaceutical Co., Ltd.; Batch Number (LOT No.): 2024/06/30]. After 4 weeks of dietary intervention (weeks 6–9), the rats were weighed, and serum was collected for ELISA analysis to measure TNF-α and IL-6 expression. Fecal samples were collected for 16S rDNA sequencing and short-chain fatty acid analysis (Figure 2).

The rats were euthanized, and their body weight and body length (from the nose to the anus) were measured. The abdominal cavity was opened to examine the size and morphology of the endometriotic lesions. The length, width, and height of the lesions were measured using a caliper, and the volume of the endometriotic lesions was calculated.

### Animal randomization and blinding procedures

Animals were randomly allocated to experimental groups using a computer-generated sequence. Where applicable, investigators involved in outcome assessments (e.g., histological scoring) and laboratory analyses (e.g., 16S rRNA gene sequencing, GC-MS, and ELISA) were blinded to group identity during data acquisition and initial analysis.

### DNA extraction and sequencing

Genomic DNA was extracted from each sample and used as the template for PCR amplification. The bacterial 16S rRNA gene *V4* hypervariable region was amplified using *barcoded primers 515F and 806R*. Sequencing libraries were constructed using a PCR-free library preparation workflow and sequenced on an *Illumina NovaSeq platform* with paired-end reads.

### Sequence processing and feature table construction

Demultiplexing was performed according to barcode sequences, and barcode/primer sequences were removed prior to downstream processing. Raw reads were quality-filtered using *fastp*, including adapter trimme ing; removal of reads containing ambiguous bases (N); filtering out reads in which >40% of bases had quality scores < 15; sliding-window trimming (4 bp window; reads were truncated when the mean quality score within the window was < 20); polyG tail trimming; and discarding reads shorter than 150 bp. High-quality paired-end reads were merged using *FLASH* to generate clean tags, and chimeric sequences were identified and removed using *vsearch*, producing effective tags for downstream analyses.

Amplicon sequence variants (ASVs) were inferred in *QIIME2* using *DADA2*; alternatively, for OTU-based analyses, sequences were clustered into OTUs at *97% sequence identity* using *UPARSE* (USEARCH v7). Low-abundance features were filtered using a minimum abundance threshold corresponding to *0.005%* of total tags after rarefaction.

### Taxonomic assignment and normalization

Taxonomic assignment was performed using a *mothur-based* classification approach against the *SILVA SSU rRNA* reference database (*SILVA v138.1*), with a confidence threshold of *0.8–1.0*. Representative sequences were aligned using *MAFFT* and used for phylogenetic analyses. To account for unequal sequencing depth across samples, the feature table was *rarefied to the minimum sequencing depth* prior to downstream alpha- and beta-diversity analyses and related community comparisons.

### PICRUSt2 functional prediction

Functional profiles were inferred from the 16S-derived feature table using *PICRUSt2 (v2.5.3)*. Predicted functional annotations were summarized using *KEGG*-based outputs. Normalized KEGG abundance tables were used for downstream community-level comparisons. Because PICRUSt2 infers functional potential from 16S profiles rather than directly measuring metagenomes, functional results were interpreted as **predicted** functional potential and reported accordingly.

### Co-occurrence network analysis and robustness assessment

Microbial co-occurrence networks were inferred using Spearman correlations implemented in microeco (trans_network, cor_method = “spearman”). To reduce sparsity-related artifacts, taxa were pre-filtered using a global relative-abundance threshold (filter_thres = 0.001) and an additional prevalence filter requiring presence in at least 10% of samples (prevalence_thres = 0.10). Pairwise associations were retained if |ρ| > 0.30 and the Benjamini–Hochberg False Discovery Rate (FDR)-adjusted q-value was < 0.05. Networks were constructed as undirected weighted graphs using igraph, with edge weights defined as |ρ|; the sign of each association (positive/negative) was retained separately for annotation and visualization. We additionally performed a sensitivity analysis across multiple correlation thresholds (|ρ| = 0.15–0.50) to evaluate the dependence of global network descriptors on the cutoff choice.

To assess robustness under limited sample sizes, we performed sample bootstrapping (*B* = 200) to estimate edge stability (edge support proportion) and hub stability (frequency of taxa appearing among the top-degree nodes). A consensus edge set was defined by retaining edges with bootstrap support ≥0.70. We also conducted a permutation-based null analysis (*P* = 50) by permuting sample order within each taxon to generate a null distribution of edge counts under no cross-taxon association; empirical *p*-values were computed by comparing observed connectivity to the null distribution. Network connectivity metrics are reported as structural descriptors and are not interpreted as ecological “stability” unless supported by explicit quantitative robustness/stability measures.

### Statistical analysis

Statistical analyses were performed using SPSS v23.0, and figures were generated using GraphPad Prism v10.4 and R v4.3.1. Normality was assessed using the Shapiro–Wilk test and homogeneity of variance using Levene's test. For two-group comparisons (NCD vs. NCD_Pro), a two-tailed Student's t-test was used for approximately normally distributed data with equal variances; Welch's t-test was applied when variances were unequal. When normality assumptions were not met, the Mann–Whitney U test was used. Data are presented as mean ± SEM for approximately normally distributed variables; otherwise, results are summarized as median (IQR). Statistical significance was defined as *P* < 0.05 (two-sided), unless otherwise specified.

For microbiome analyses, microbial community profiling, diversity analyses, ordination, and differential feature detection were conducted in R v4.3.1 using the microeco package. Briefly, a microeco microtable object was constructed from the feature table (ASV/OTU table), sample metadata, and taxonomy table, and the dataset was standardized using the built-in data tidying procedures. Alpha diversity indices (e.g., Chao1, Shannon, and Pielou) were calculated using microeco's alpha-diversity module (e.g., trans_alpha) and compared between groups using the two-group testing strategy described above. Beta diversity was evaluated using UniFrac distance matrices and visualized by PCoA using microeco's beta-diversity/ordination module (e.g., trans_beta); group separation was tested by PERMANOVA implemented in microeco with 999 permutations (two-sided, permutation-based significance).

Differential microbial features were identified using LEfSe implemented in microeco (e.g., trans_diff with method = “lefse”), using a significance level of α = 0.05 and an Linear Discriminant Analysis (LDA) score cutoff of 4.0 for microbial taxa (unless otherwise stated in figure legends). For PICRUSt2-predicted functional profiles, differential KEGG features were assessed using LEfSe with an LDA score cutoff >3.5 as specified in the analysis settings. For analyses involving multiple comparisons (e.g., taxa/pathway screening and correlation matrices), *p*-values were adjusted using the Benjamini–Hochberg false discovery rate (FDR) and q-values are reported where applicable; statistical significance after correction was defined as *q* < 0.05.

## Results

### Probiotic treatment can reduce TNF-α and IL-6 levels in EMs

**Figure 3:**
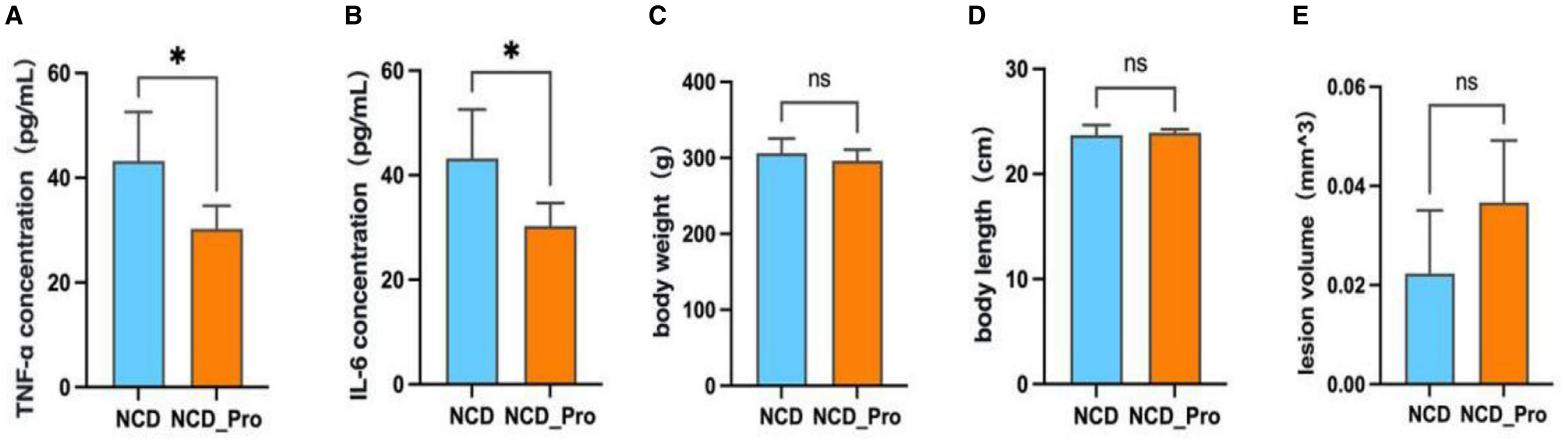
**(A)** Serum levels of TNF-α following different diet treatments. **(B)** Serum levels of IL-6 following different diet treatments. **(C)** Comparison of body weight changes after different diet treatments. **(D)** Comparison of body length changes after different diet treatments. **(E)** Comparison of endometriotic lesion volumes after different diet treatments in rats. **(A–E)** Bar graphs depict mean + SEM, 4 rats/group, pairwise comparisons by Kruskal-Wallis rank-sum test.

Compared with the NCD group, the levels of pro-inflammatory cytokines TNF-α and IL-6 in the serum of NCD_Pro rats were significantly reduced (*P* < 0.05; Figures 3A, B). However, no significant differences were observed in body weight, body length, or lesion volume between the two groups (*P* > 0.05; Figures 3C–E).

### Diversity analysis between NCD and NCD-pro

**Figure 4:**
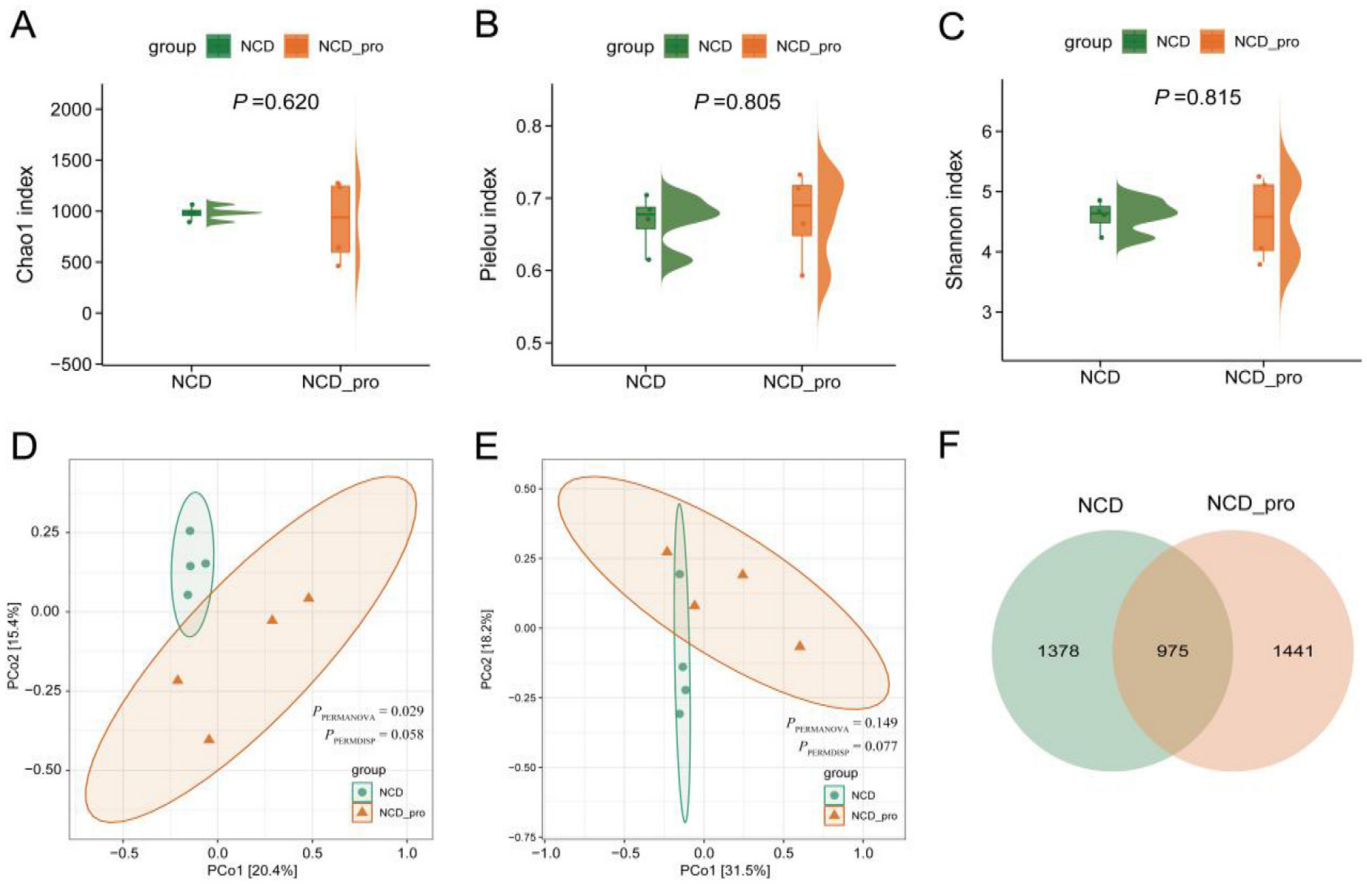
Differences in alpha diversity indices between NCD and NCD_Pro groups. Violin plots showing the distribution of **(A)** Chao1 index, **(B)** Pielou index, and **(C)** Shannon index in NCD and NCD_Pro groups, with *P*-values indicating no significant differences between the groups. Principal Coordinate Analysis (PCoA) plots based on **(D)** Unweighted UniFrac distance and **(E)** Weighted UniFrac distance. **(F)** Venn diagram showing the number of OTUs shared and unique between NCD and NCD_Pro groups. Comparisons of Chao1 and Pielou between the NCD and NCD_Pro groups were analyzed using the Kruskal-Wallis rank-sum test.

In this study, we compared microbial richness and diversity between the NCD and NCD-Pro groups. Alpha diversity was evaluated using the Chao1, Pielou, and Shannon indices, which revealed no significant differences between the two groups (*P* > 0.05; Figures 4A–C). For beta diversity, unweighted UniFrac analysis demonstrated a significant difference between NCD and NCD-Pro (*P* < 0.05; Figure 4D), whereas weighted UniFrac analysis showed no significant difference (*P* > 0.05; Figure 4E). Furthermore, a Venn diagram was used to visualize the shared and unique bacterial operational taxonomic units (OTUs) among the groups. As shown in Figure 4F, the NCD-Pro group exhibited a richer bacterial community composition, with 975 OTUs common to both groups (Figure 4F). Together, these findings suggest that probiotic administration modulated the gut microbiota composition in rats.

### Bacteria composition and relative abundance

**Figure 5:**
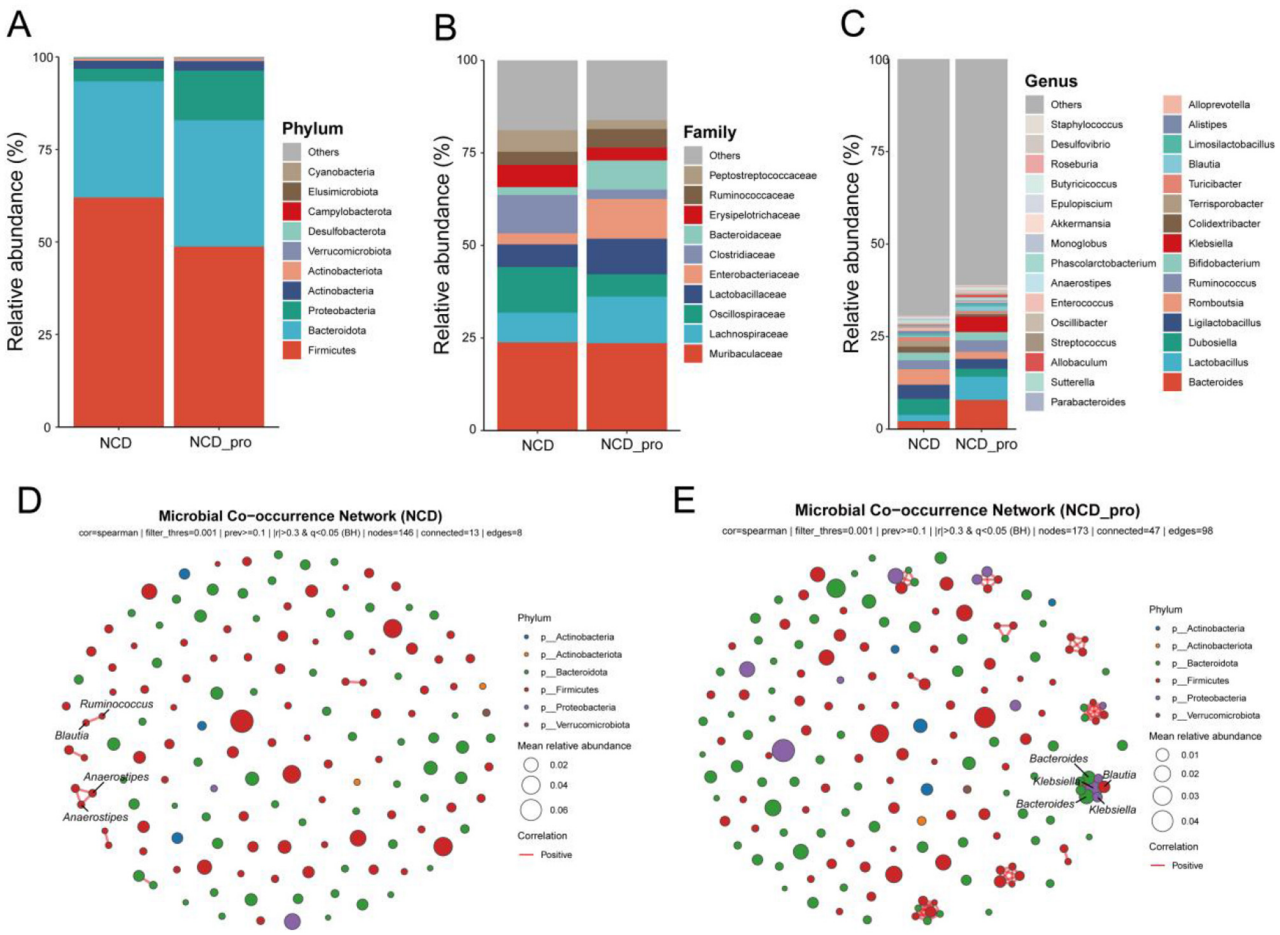
Relative abundance of gut microbiota at different taxonomic levels in NCD and NCD_Pro groups. Stacked bar plots showing the distribution at the **(A)** phylum level, **(B)** family level, and **(C)** genus level. Microbial co-occurrence network analysis in **(D)** NCD group and **(E)** NCD_Pro group.

From all fecal samples, we chose the main phylum, family and genus based on species abundance to generate a histogram, which showed the percentages of relative abundance in each group (Figures 5A, B).

At the phylum level (Figure 5A), the top three most abundant phyla were Firmicutes, Bacteroidota, and Proteobacteria. Compared with the control group (NCD), the relative abundance of Firmicutes was lower in the probiotic intervention group (NCD_Pro), while the relative abundances of Bacteroidota and Proteobacteria were higher. At the family level (Figure 5B), distinct differences in the relative abundance of gut microbiota were observed between the two groups. In the NCD group, the most abundant families were Muribaculaceae, Oscillospiraceae, and Clostridiaceae, whereas in the NCD_Pro group, Muribaculaceae, Lactobacillaceae, and Enterobacteriaceae showed the highest abundance. Notably, certain bacterial families exhibited an inverse abundance pattern between the two groups—those with higher relative abundance in the NCD_Pro group were significantly reduced in the NCD group. At the genus level, Bifidobacterium and Lactobacillus were enriched in the NCD_Pro group but were not predominant in the NCD group. Moreover, Klebsiella and Allobaculum were scarcely detected or virtually absent in the NCD group. Notably, the increased relative abundance of the genus Klebsiella in the probiotic group warrants cautious interpretation, because this genus includes opportunistic pathogens. However, the overall relative abundance of Klebsiella remained low in both groups, no clinical signs of infection or systemic toxicity were observed, and 16S rDNA sequencing at the genus level does not allow discrimination between potentially pathogenic and non-pathogenic species (Figure 5C).

Microbial co-occurrence network analysis further indicated that the microbial network in the probiotic intervention group (NCD_Pro) had higher numbers of nodes and connections than the control group (NCD; Figures 5D–E), suggesting that probiotic intervention enhanced the interactions among various members of the gut microbiota. In the co-occurrence network of the NCD group, the core genera were Anaerostipes, Ruminococcus, and Blautia; whereas in the NCD_Pro group, the core genera were Bacteroides, Klebsiella, and Blautia.

### Bacterial composition difference analysis

**Figure 6:**
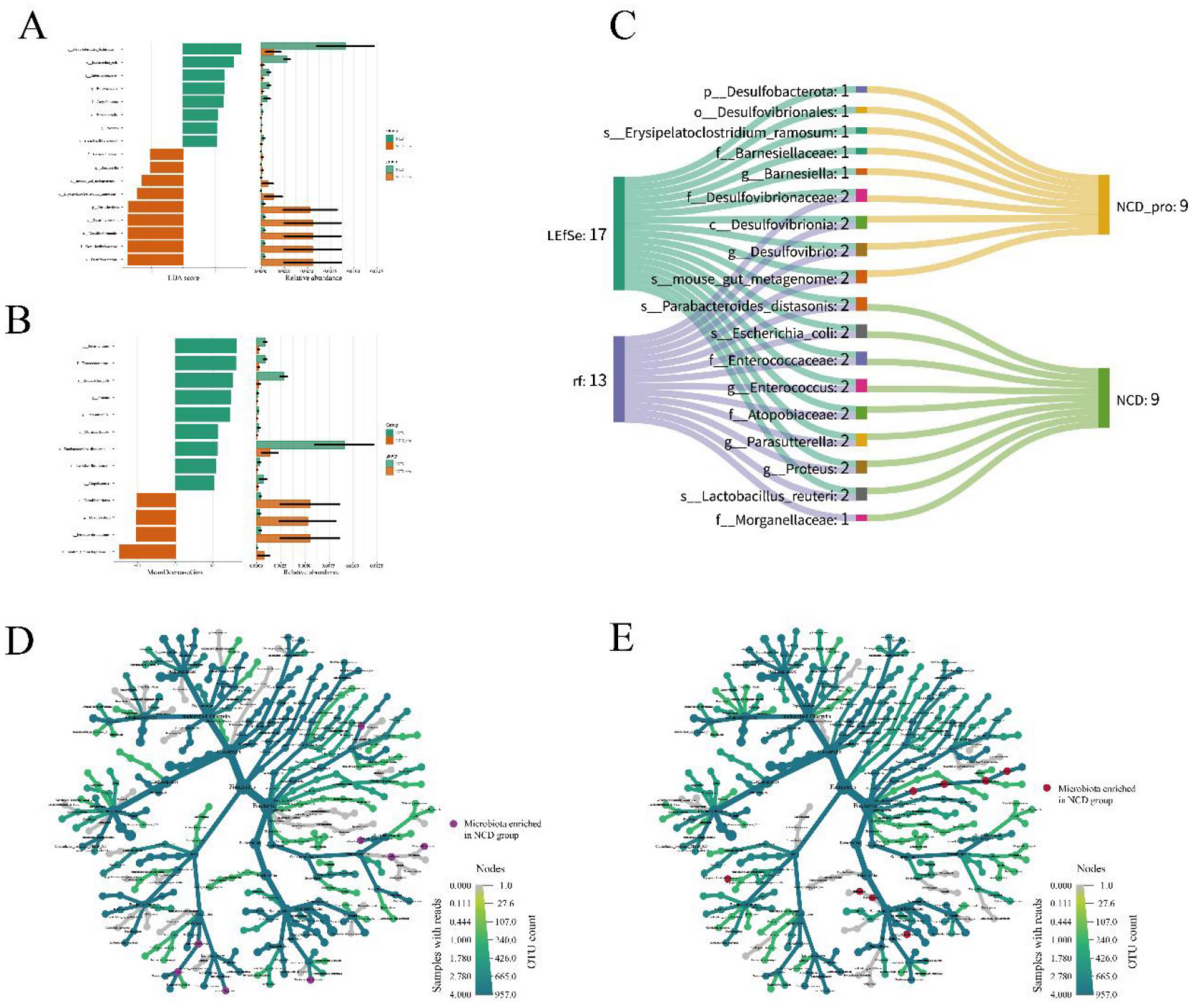
Differentially abundant microbial taxa identified by **(A)** LEfSe and **(B)** Random Forest methods. The left panels show the taxonomic cladogram highlighting the microbial taxa with significant differences between NCD and NCD_Pro groups. The right panels display the relative abundance of these taxa in each group. **(C)** Sankey diagram illustrating the distribution of differentially abundant microbial taxa between NCD and NCD_Pro groups. The width of the bands represents the relative abundance of the taxa. Microbial interaction networks in **(D)** NCD group and **(E)** NCD_Pro group. Nodes represent microbial taxa, and edges represent significant interactions between them. The size and color of the nodes correspond to the OTU count and the phylum of the taxa, respectively. Microbiota enriched in NCD and NCD_Pro groups are marked in purple and red, respectively.

To identify the key differential microbial taxa caused by probiotic intervention, this study used LEfSe analysis and the Random Forest algorithm for feature selection and importance assessment. LEfSe identified 17 differential taxa, with eight enriched in the NCD group and nine in the NCD-pro group. Random Forest identified 13 differential taxa, with nine enriched in the NCD group and four in the NCD-pro group. A Sankey diagram further intuitively displayed the distribution of differential taxa between the two groups (Figures 6A–C). From the diagram, it can be seen that both analysis methods consistently identified 18 differential taxa. Among them, c_Desulfovibrionia, g_Desulfovibrio, f_Desulfovibrionaceae, and g__Barnesiella were enriched in the NCD_Pro group, indicating that sulfate-reducing bacteria, including Desulfovibrio, are among the taxa most strongly affected by the probiotic intervention. Given the reported association of these bacteria with both homeostatic and pro-inflammatory states, their enrichment should be interpreted with caution. In contrast, pathogenic taxa such as s_parabacteroides_distasonis and s_Escherichia_coli were enriched in the NCD group, indicating that probiotic intervention may have suppressed the abundance of these potential pathogens to some extent.

To further assess the impact of probiotic intervention on microbial interaction patterns, we performed a co-occurrence network analysis. The results revealed that, compared to the NCD group (purple nodes), the probiotic group exhibited a marked increase in connection density among dominant bacterial taxa (red nodes), suggesting enhanced interspecific cooperation and improved ecological network stability (Figures 6D, E). Furthermore, as illustrated in Figures 5F, G, the NCD_Pro microbiota displayed greater complexity, with significantly elevated network density and connectivity, indicating that probiotic administration effectively promoted synergistic interactions among gut microbes and reinforced ecological stability.

### Analysis of functional differences in microorganisms

**Figure 7:**
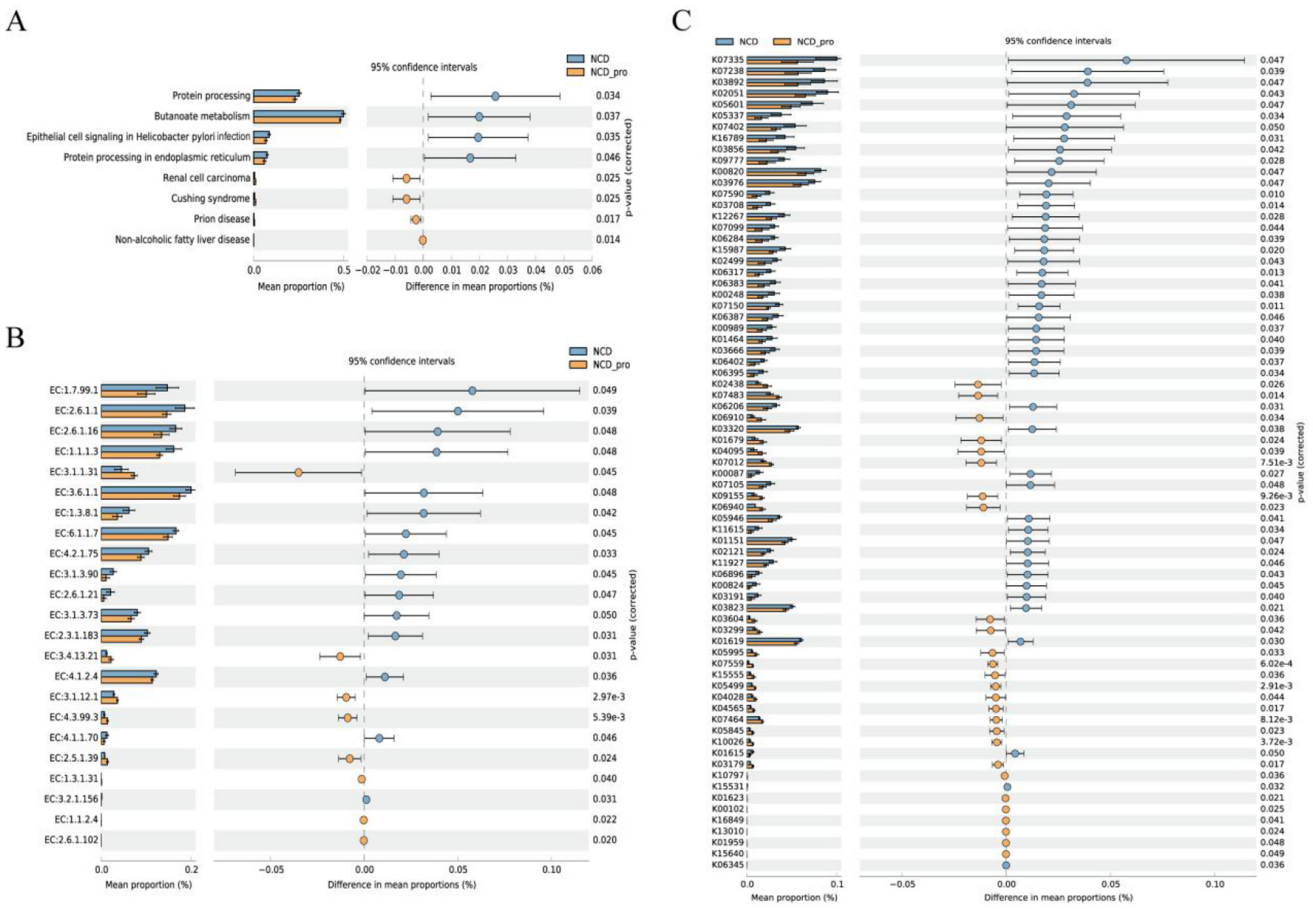
PICRUSt2-predicted differential functional profiles. **(A)** Differentially enriched MetaCyc metabolic pathways; **(B)** differential abundance of enzyme functions (EC numbers); **(C)** differential KEGG Orthology (KO) functions.

In this study, the PICRUST2 software was used to predict the functional abundance of the gut microbiota and obtain information on bacterial pathways, EC metabolic enzymes, and KO pathways. The LEfSe algorithm was applied with an LDA threshold of >3.5 to analyze the differences in microbial functions between the two groups. Pathway analysis showed that Protein processing, Butanoate metabolism, Epithelial cell signaling in Helicobacter pylori infection, and Protein processing in endoplasmic reticulum were more abundant in the NCD group, while Renal cell carcinoma, Cushing syndrome, and Non-alcoholic fatty liver disease were more abundant in the NCD_Pro group (Figure 7A). EC enzyme analysis revealed that 15 metabolic enzymes, including EC:1.7.99.1, EC:2.6.1.1, and EC:2.6.1.16, were more abundant in the NCD group, whereas 8 metabolic enzymes, such as EC:3.1.1.31, EC:3.4.13.21, EC:3.1.12.1, and EC:4.3.99.3, were more abundant in the NCD_Pro group (Figure 7B). KO pathway analysis indicated that pathways such as KO7335, KO7238, and KO3892 were more abundant in the NCD group, while pathways like KO2438, KO7483, and KO6910 were more abundant in the NCD_Pro group (Figure 7C). It should be emphasized that these disease-labeled KEGG pathways are derived from a predictive bioinformatics algorithm and most likely reflect enrichment of shared underlying metabolic modules rather than actual occurrence of renal cell carcinoma, Cushing syndrome, or non-alcoholic fatty liver disease in our rat model.

### Unique bacterial functional characteristics between NCD and NCD_Pro

**Figure 8:**
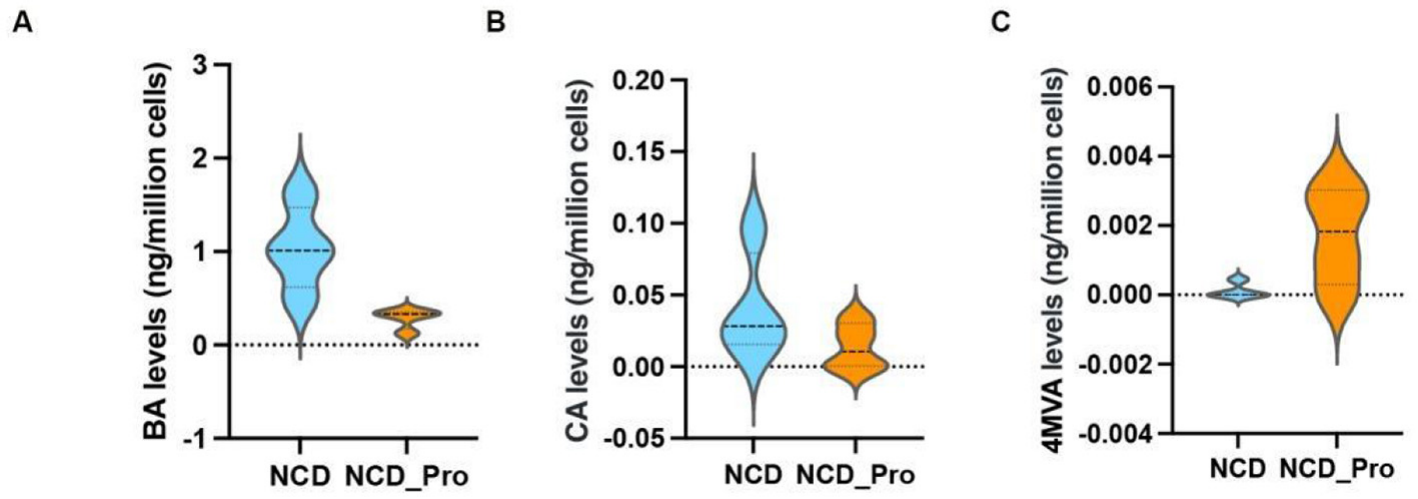
**(A)** Difference in BA levels between the different groups. **(B)** Difference in CA levels between the different groups. **(C)** Difference in 4-MVA levels between the different groups. Comparisons between the NCD and NCD_Pro groups were analyzed using the Kruskal-Wallis rank-sum test.

**Figure 9:**
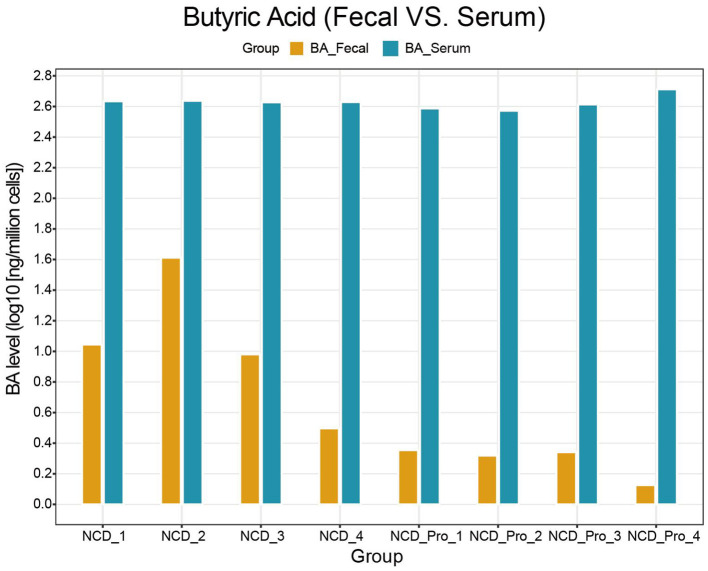
Difference in BA levels between fecal and serum.

**Figure 10:**
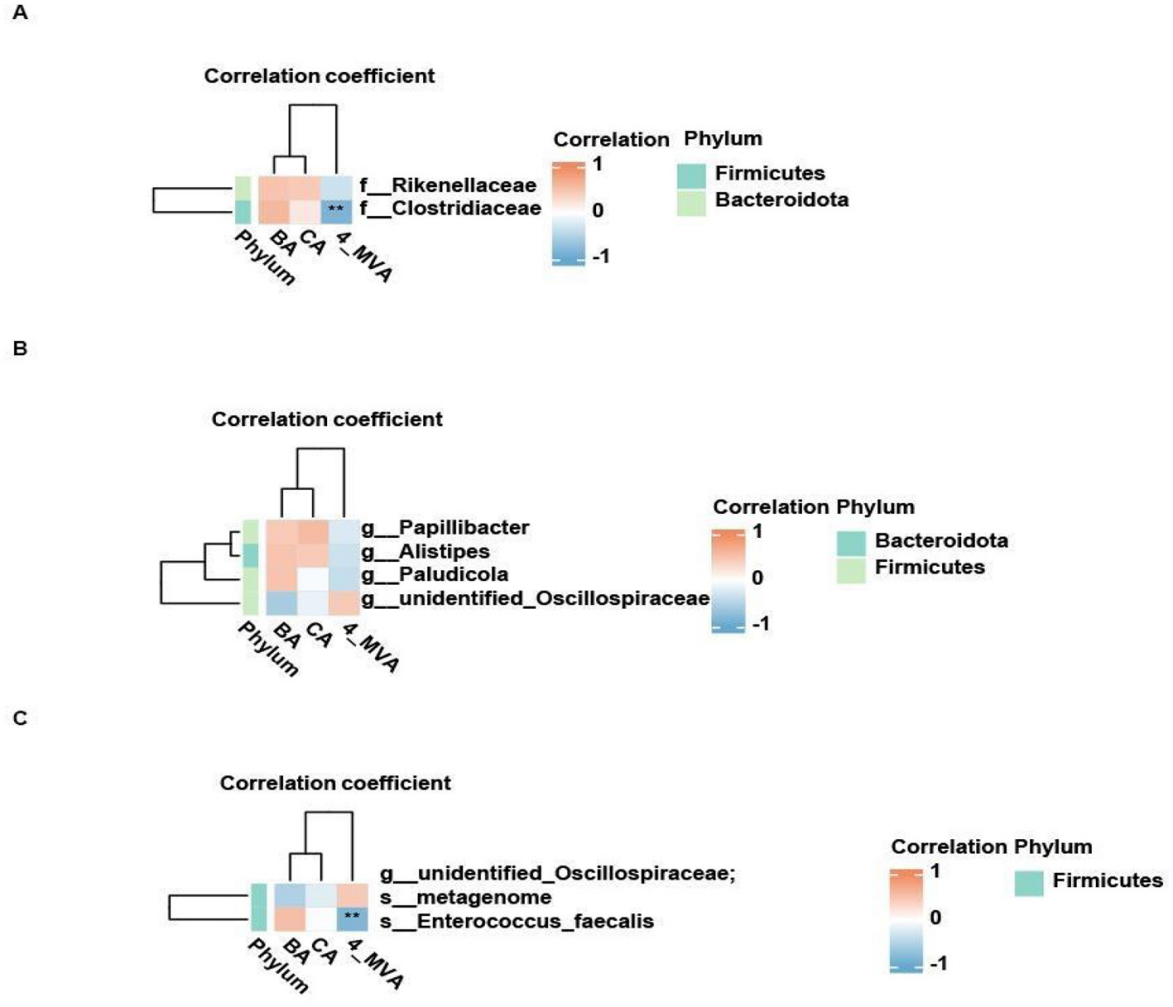
**(A)** Relationship between differentially abundant species at the family level and differential metabolites. **(B)** Relationship between differentially abundant species at the genus level and differential metabolites. **(C)** Relationship between differentially abundant species at the species level and differential metabolites. Rows represent microorganisms, and columns represent metabolites. The evolutionary tree on the left shows the microbial hierarchical clustering results, while the evolutionary tree at the top shows the hierarchical clustering results for metabolites. Red indicates a positive correlation, and blue indicates a negative correlation. *denotes a significant difference (*P*-value < 0.05), **denotes a highly significant difference (*P*-value < 0.01).

We measured the concentrations of SCFAs in feces and identified three metabolites that showed significant differences between the two groups: BA (butyrate), CA (caproate), and 4-MVA (isocaproate; Table 1). As shown in Table 1, probiotic intervention significantly altered the fecal SCFA profile in rats: compared with the NCD group, the NCD_Pro group had significantly lower concentrations of BA and CA, while the level of 4-MVA was markedly increased (Figures 8A–C). However, this study further revealed that serum BA levels were significantly higher than fecal BA levels, suggesting that fecal BA cannot be equated with bioavailable BA in the body (Figure 9). Spearman correlation analysis further revealed that the differentially enriched Clostridiaceae and Enterococcus faecalis were significantly negatively correlated with 4-MVA (*r* < −0.6, ***P*** < 0.01; Figures 10A–C). The above results suggest that probiotics can reshape the gut microbiota–metabolite axis to modulate the SCFA microenvironment in rats with endometriosis.

**Table 1:** Screening of differential metabolites.

**Index**	**Compounds**	**Class**	**Fold_change**	**Type**
BA	Butyric acid	SCFA	0.27596907590426234	Down
CA	Caproic acid	SCFA	0.27596907590426234	Down
4-MVA	Isocaproic acid	SCFA	15.4593194512612	Up

Select metabolites with a fold change ≥2 and fold change ≤ 0.5. Metabolites that show a difference of more than 2-fold or less than 0.5-fold between the control and experimental groups are considered significantly different. “Up” indicates upregulation, and “down” indicates downregulation.

**Figure 11:**
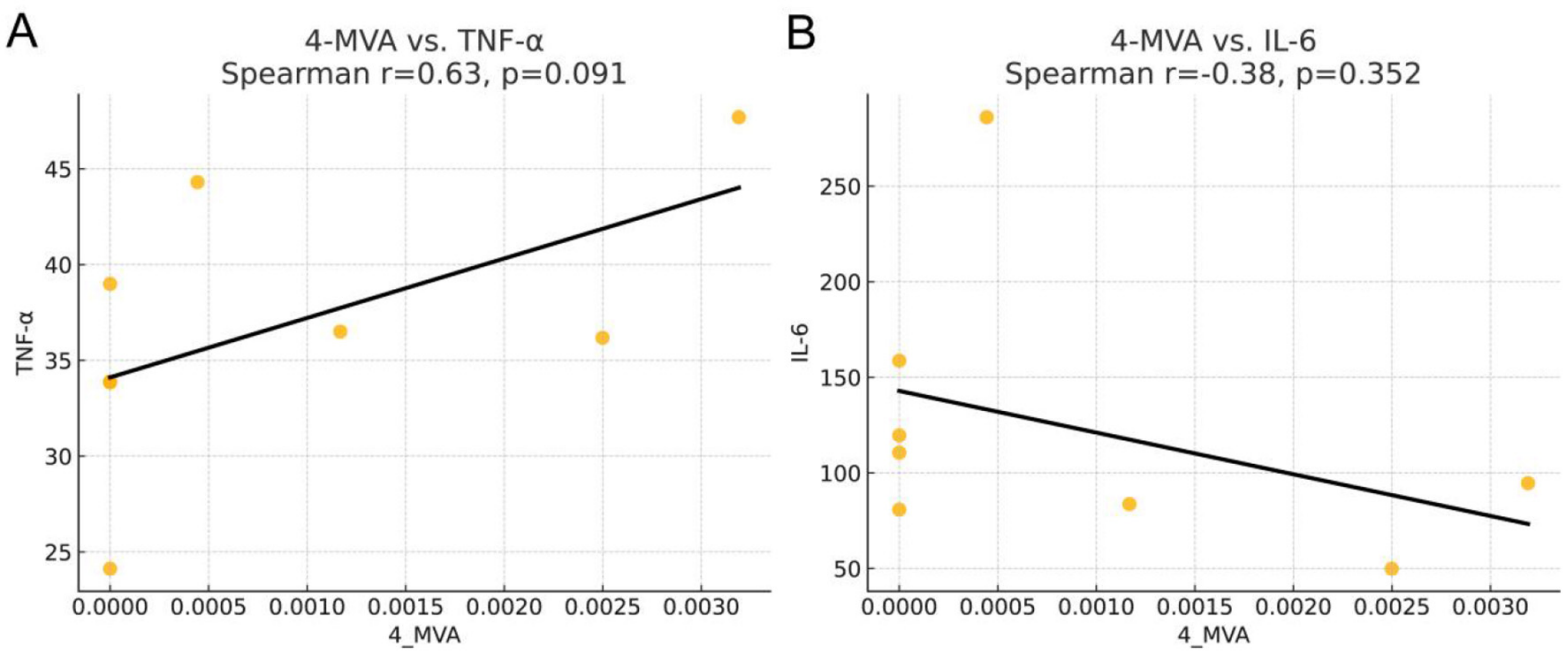
Correlation between fecal isocaproic acid (4-MVA) levels and systemic inflammatory markers. **(A)** 4-MVA vs. TNF-α shows a moderate positive correlation (Spearman *r* = 0.63, *P* = 0.091). **(B)** 4-MVA vs. IL-6 shows a weak negative correlation (Spearman *r* = −0.38, *P* = 0.35). Fecal 4-MVA levels and systemic inflammatory markers were assessed by Spearman correlation analysis.

As shown in the Figure 11, 4-MVA exhibited a moderate positive correlation with TNF-α (Spearman *r* = 0.63, *P* = 0.091) and a weak negative correlation with IL-6 (Spearman *r* = −0.38, *P* = 0.352); However, neither association reached statistical significance, suggesting that any potential relationship between 4-MVA and these inflammatory cytokines remains tentative and warrants further investigation in larger samples.

## Discussion

This study established a rat model of endometriosis (EMs) to evaluate the impact of probiotic dietary intervention on gut microbiota composition and systemic inflammatory response. Comprehensive analysis of the gut microbiota using 16S rRNA sequencing technology revealed that probiotic intervention significantly reshaped the gut microbial community structure, particularly altering the abundance of specific dominant bacterial species. Although no significant differences in α-diversity were observed between the NCD group and the NCD_Pro group, β-diversity analysis indicated a significant separation in microbial structure between the two groups, suggesting that probiotic intervention may modulate the overall composition of the gut microbiota. In addition, probiotic intervention significantly reduced the systemic inflammation levels in the model animals and significantly reshaped the gut microbial functions and their metabolic profiles. These findings suggest that probiotic intervention may influence inflammatory status by regulating the structure and function of the gut microbiota.

### Probiotic intervention reduced the level of systemic pro-inflammatory cytokines (TNF-α, IL-6)

Inflammatory response plays a key role in the pathogenesis and progression of endometriosis (Greygoose et al., 2025). TNF-α and IL-6 are key molecules mediating EMs-related inflammation and pain, and their decreased levels may exerte systemic anti-inflammatory effects. This study found that probiotic intervention significantly reduced the levels of pro-inflammatory cytokines TNF-α and IL-6 in rat serum. Notably, this anti-inflammatory effect was not significantly associated with changes in animal body weight, body length, or the volume of endometriotic lesions, suggesting that probiotics do not directly inhibit the growth of ectopic lesions but may exert anti-inflammatory effects through other pathways. The findings of this study align with those reported by Arthur et al. (2013), suggesting that probiotics may primarily modulate or stabilize the composition of the mucosal microbiota and restore mucosal barrier function, thereby influencing levels of related inflammatory factors, rather than directly reversing established tumor masses (Ma et al., 2004; Wang et al., 2019). Furthermore, a fecal microbiota transplantation study utilizing Lactobacillus strains isolated from chemotherapy-responsive patients demonstrated that while such strains exhibit significant anti-inflammatory effects, they do not affect the efficacy of the antitumor therapy itself (Yu et al., 2025). Together, this evidence indicates that the role of probiotics in the tumor microenvironment may lean more toward providing “mucosal protection and immunomodulation” rather than directly killing tumor cells. Further analysis indicated that probiotic intervention altered the gut microbiota structure, suggesting that its anti-inflammatory mechanism may be related to the regulation of gut microbial composition. This is consistent with the previous view that probiotics alleviate chronic inflammatory diseases by modulating the gut microbiota structure (Cao et al., 2023; Zhou et al., 2022). Future studies with larger sample sizes and additional local inflammatory readouts (e.g., lesion histology scoring, immune cell profiling, or peritoneal fluid cytokine analysis) are warranted to validate the anti-inflammatory effects of probiotics.

### Probiotics alter gut microbiota structure and network stability

To explore the mechanism by which probiotics alter gut microbiota structure to exert anti-inflammatory effects, we analyzed the changes in the gut microbiota. Although there were no significant differences in α-diversity indices between the two groups, β-diversity analysis based on unweighted UniFrac distance showed a significant separation in microbial structure between the two groups, indicating that probiotics can precisely alter the abundance of specific bacterial populations without disrupting the overall diversity structure. The review by Iavarone et al. (2023) demonstrated that the ratio of Firmicutes to Bacteroidota is increased in patients with endometriosis compared to controls, a finding that aligns with our results. At the phylum level, probiotic intervention led to a decrease in the relative abundance of Firmicutes, while the abundance of Bacteroidota, and Proteobacteria increased. Previous studies have indicated that the Firmicutes/Bacteroidota ratio (F/B ratio) is often significantly elevated in pathological conditions such as enteritis or constipation and is closely related to inflammatory activity. The observed decrease in the F/B ratio in this study further supports the inference that probiotics may alleviate inflammation by regulating this ratio and may affect host immune function. In addition, at the genus level, significant enrichment of Lactobacillus and Bifidobacterium was observed in the probiotic intervention group, providing further evidence for the direct anti-inflammatory effects of probiotic intervention. Previous studies have shown that lactobacilli can enhance the complexity of the gut microbiota and have beneficial effects on metabolism and immune regulation (Pessione, 2012). A recent review further concluded that oral probiotics can alleviate endometriosis-related symptoms, as evidenced by increased IL-12 levels and reduced natural killer (NK) cell activity, suggesting an immunomodulatory role of probiotics in this disease context (Iavarone et al., 2023). The potential mechanism may involve Lactobacillus promoting the production of SCFAs, enhancing intestinal mucosal barrier function, and stimulating the expression of anti-inflammatory factors, thereby synergistically inhibiting inflammatory responses (Partrick et al., 2021; Wang et al., 2022b). More interestingly, microbial co-occurrence network analysis revealed that the number of microbial network nodes and connectivity were significantly higher in the probiotic intervention group than in the control group, and the core genera also changed. This indicates that probiotics not only introduce or proliferate specific beneficial bacteria but also promote more intimate and complex interactions among microbial members, thereby enhancing the stability and synergistic capabilities of the entire microbial ecosystem. A more stable and diverse microbial network may be more resistant to dysbiosis under pathological conditions. From a functional perspective, the shift of core genera from Anaerostipes/Ruminococcus/Blautia in the control network to Bacteroides/Klebsiella/Blautia in the probiotic group may reflect a reorganization of carbohydrate- and amino-acid-fermenting niches. Bacteroides are efficient degraders of complex polysaccharides and may provide substrates (e.g., acetate, lactate, and succinate) that are further utilized by other SCFA-producing taxa, whereas Blautia has been associated with butyrate and acetate production. The increased connectivity of Klebsiella within this network could indicate a role in cross-feeding or niche occupation under probiotic intervention, but its net impact on host inflammation remains unclear and requires further mechanistic studies.

### Changes in key differential microbiota and functional pathways

In this study, 18 core differential microbiota were identified by the combined use of LEfSe and RF, among which it is noteworthy that the NCD-pro group significantly enriched sulfate-reducing bacteria (SRB), including the class c_Desulfovibrionia, genus g_Desulfovibrio, and family f_Desulfovibrionaceae. SRB are anaerobic, Gram-negative, symbiotic residents in the colons of humans and other animals and are abundant in inflammatory diseases such as inflammatory bowel disease (IBD), colitis, pouchitis, and periodontitis (Gibson et al., 1988; Singh and Lin, 2015). The traditional view is that the H_2_S generated by SRB mainly exerts colonic mucosal toxicity by inhibiting cytochrome c oxidase, disrupting the epithelial barrier, and inducing inflammatory responses (Figliuolo et al., 2018; Singh et al., 2021). However, an increasing amount of evidence suggests that the biological effects of H_2_S have a “dual nature”: as an endogenous gaseous signaling molecule listed alongside nitric oxide and carbon monoxide, low concentrations of H_2_S can activate ATP-sensitive potassium (KATP) to mediate vasodilation of vascular smooth muscle, increase blood flow at the ulcer margin, and thereby promote colonic mucosal repair and tissue homeostasis maintenance (Singh et al., 2021; Zhao et al., 2001). Moreover, studies have shown that an appropriate amount of Desulfovibrio can form a symbiotic relationship with Faecalibacterium prausnitzii, with F. prausnitzii producing lactate as a substrate and Desulfovibrio consuming lactate and reducing sulfate, ultimately promoting butyrate production in the gut (Khan et al., 2023). Butyrate is the main energy source for colonic epithelial cells and has anti-inflammatory and barrier-protective functions (Louis and Flint, 2017; Peng et al., 2022). Taken together, our data indicate that probiotic intervention reshaped the gut ecosystem in a way that included an enrichment of Desulfovibrio. Because sulfate-reducing bacteria have been linked to both detrimental mucosal toxicity and potential homeostatic functions, the net impact of this shift on host inflammation remains uncertain. Importantly, we did not directly quantify H_2_S production or assess mucosal barrier integrity in this study; therefore, any putative beneficial effect of Desulfovibrio should be regarded as speculative. Future work should directly measure luminal and mucosal H_2_S levels, as well as barrier-related endpoints, to clarify whether the observed changes in SRB are protective or harmful in the context of endometriosis. In addition, this study found that g__Barnesiella was enriched in the NCD_Pro group. Some studies have shown that g__Barnesiella can promote anti-tumor immune responses (Daillère et al., 2016; Yan et al., 2022).

The enrichment of Butanoate metabolism in the NCD group suggests that the gut microbiota in the un-intervention state may maintain mucosal barrier function through butyrate synthesis. The decreased abundance of this pathway after probiotic intervention may reflect changes in microbial structure and a reduction in butyrate-producing bacteria. The enrichment of Epithelial cell signaling in Helicobacter pylori infection in the NCD group suggests the presence of inflammation in the un-intervention group, consistent with our results on pro-inflammatory cytokine levels. The cag pathogenic island of Helicobacter pylori can activate the NF-κB pathway through the Nod1 receptor, driving epithelial cell inflammatory responses, and long-term infection is associated with an increased risk of gastric cancer (Viala et al., 2004). The decreased abundance of this pathway after probiotic intervention may be achieved through competitive inhibition of Helicobacter pylori colonization or modulation of immune responses to exert a protective effect.

Notably, the PICRUSt2-based predictions that the probiotic group was enriched in disease-labeled KEGG pathways such as “Renal cell carcinoma”, “Cushing syndrome”, and “Non-alcoholic fatty liver disease” should be interpreted with great caution. KEGG disease categories are often driven by combinations of fundamental metabolic modules (e.g., cell proliferation, lipid metabolism, and redox balance) that are shared across multiple conditions. In our study, there is no histological or clinical evidence of these diseases, and the animal model was not designed to induce them. We therefore interpret these pathway enrichments as algorithm-derived, exploratory indicators of shifts in underlying microbial metabolic potential, rather than as markers of disease risk.

### Regulation of the gut microbiota-metabolite axis: the complex role of short-chain fatty acids (SCFAs)

The gut microbiota interacts with the host through its metabolites, among which SCFAs are key signaling molecules. SCFAs, key metabolites produced by specific groups of gut microbiota, play a pivotal role in maintaining gut homeostasis. Deficiencies in SCFAs have been linked to the pathogenesis of various diseases, including inflammatory bowel disease, colorectal cancer, and cardiometabolic disorders. The synthesis of these crucial molecules is directly influenced by the availability of specific dietary substrates, particularly prebiotics, through dietary or supplemental interventions (Fusco et al., 2023). The production of SCFAs, notably butyrate (BA), is closely associated with bacterial taxa within the phylum Firmicutes (Fusco et al., 2023). This study found that probiotic intervention significantly altered the SCFA profile in rat feces: the concentrations of BA and caproate (CA) were significantly reduced, while the level of isocaproate (4-MVA) was significantly increased. The decreased relative abundance of *Firmicutes* in the NCD_Pro group may account for the concurrent reduction in the levels of BA. This result presents a complexity worth further discussion. On the one hand, BA, as one of the most important SCFAs, is generally considered anti-inflammatory (Recharla et al., 2023). Its decrease in the probiotic group seems to contradict the result of reduced inflammation. On the other hand, correlation analysis suggested that the Lactobacillus genus, which was enriched in the probiotic group, was positively correlated with butyrate levels. This seemingly contradictory phenomenon may reflect the complexity of SCFA metabolism: the final concentration of SCFAs in feces is the net result of the entire microbiota's production and consumption, and the contribution of a single genus may be overshadowed by the overall effect of other microbial members (such as changes in the main butyrate-producing genus Clostridium). From the classical viewpoint that higher BA production is generally beneficial, the observed decrease in fecal BA in the probiotic group appears counterintuitive and contrasts with the common perception that probiotics invariably increase butyrate. However, the relationship between probiotic supplementation, SCFA profiles, and host inflammation is highly context-dependent and influenced by the specific strains used, dietary background, and disease state. In our endometriosis model, the probiotic-induced reduction in systemic inflammatory markers (TNF-α and IL-6) occurred despite lower fecal BA, suggesting that the anti-inflammatory effects of the intervention are not solely mediated by increased butyrate excretion in feces. In addition, the level of SCFAs in feces may not necessarily be equivalent to their bio-effective concentrations in the intestinal mucosa or portal vein. Approximately 95% of SCFAs are absorbed by the host, with only about 5% excreted in feces (den Besten et al., 2013). The final concentration of SCFAs in feces can be influenced by multiple factors, including intestinal transit time, mucosal permeability, metabolite transport, and sample processing methods (Silva et al., 2020). As highlighted in one review, the substantial inter-individual variability in fecal SCFAs limits the ability to draw conclusions about actual intestinal SCFA metabolism and systemic availability (Hamari et al., 2025). Consistent with this, existing studies indicate that fecal levels of BAs and CAs do not correlate with their circulating concentrations. Thus, fecal SCFA levels likely represent a net balance between colonic production and absorption, rather than reflecting systemic levels (Müller et al., 2019). Therefore, this study aims to validate this point in future experimental investigations. In principle, the observed reduction in fecal butyrate could reflect either decreased microbial production, increased host absorption, or a combination of both. Our current design does not allow us to distinguish between these possibilities. Future studies could address this question by simultaneously measuring SCFA concentrations in colonic contents, mucosal tissue, and portal as well as peripheral blood, and by assessing the expression of SCFA transporters (e.g., MCT1, SMCT1) and receptors (e.g., GPR41/43) in the intestinal epithelium. Stable isotope-based tracer experiments would further help to disentangle production vs. absorption of butyrate under probiotic intervention.

Meanwhile, the significant increase in isocaproate (4-MVA), a branched-chain short-chain fatty acid, in the probiotic group is an intriguing new finding. Correlation analysis showed that 4-MVA levels were significantly negatively correlated with the Clostridiaceae family, which was enriched in the control group. The Clostridiaceae family is an important anaerobic bacterial family widely present in the human gut, capable of metabolizing dietary fiber and producing large amounts of SCFAs, such as butyrate and propionate. Members of this family play a crucial role in maintaining gut health and immune function. Previous studies have shown that the abundance of Clostridiaceae is associated with SCFA synthesis, especially butyrate, which has anti-inflammatory effects and is important for maintaining gut barrier function (Berding and Donovan, 2019; Muñiz Pedrogo et al., 2019; Qiu et al., 2024). Enterococcus faecalis is a common gut bacterium that can tolerate the low pH environment of the gut and can affect the metabolic pathways of gut microbiota involved in SCFA production (Cai et al., 2024; Jeong et al., 2019). Although Clostridiaceae and Enterococcus faecalis are associated with SCFA production, the mechanisms through which they—or probiotic interventions—might influence 4-MVA levels remain unclear. What's more, correlation analyses revealed a complex relationship between 4-MVA and inflammatory mediators: it was positively correlated with the pro-inflammatory cytokine TNF-α yet negatively correlated with IL-6. This dichotomous pattern suggests that its anti-inflammatory effect is not mediated through the uniform modulation of cytokine levels. Although no study has directly established 4-MVA as an anti-inflammatory agent, a clinical investigation in patients with mental disorders reported that plasma 4-MVA levels were positively correlated with anti-inflammatory cytokines, including IL-4, IL-33, and IL-9 (Yu et al., 2024), supporting its potential immunomodulatory role. Mechanistic insights, however, primarily come from research on its derivative, pogostone, for which 4-MVA serves as a key biosynthetic precursor (Chen et al., 2021; Wang et al., 2022a). Given the well-documented anti-inflammatory activity of pogostone, its biosynthetic link to 4-MVA provides a compelling rationale for future investigations into the direct immunomodulatory functions of the precursor molecule. In summary, the marked increase in 4-MVA, together with its significant correlations with specific differential taxa and its non-significant trends with inflammatory cytokines, suggests that 4-MVA may be a candidate metabolite involved in the probiotic modulation of the gut-immune axis. The probiotic preparation used in this study contained Bacillus subtilis and Lactobacillus acidophilus, both of which are known to modulate gut metabolism and immune responses. Lactic acid-producing lactobacilli can lower luminal pH, produce antimicrobial compounds, and provide substrates (e.g., lactate) for cross-feeding by other anaerobes, whereas Bacillus species can secrete a broad range of enzymes that facilitate the breakdown of complex dietary components. Although direct evidence linking these specific strains to Desulfovibrio expansion or 4-MVA production is currently lacking, it is plausible that they indirectly shape the gut environment-through changes in substrate availability, pH, and redox conditions-in a way that favors shifts in sulfate-reducing bacteria and branched-chain SCFAs. This interpretation remains speculative and requires targeted mechanistic studies using mono- or defined consortia colonization models. One logical next step would be to administer 4-MVA directly to EMs model rats at several doses, with appropriate vehicle and probiotic control groups, and to assess endometriotic lesion burden, systemic inflammatory markers, pain-related behaviors, and intestinal barrier function. Complementary *in vitro* studies exposing immune cells or endometrial stromal cells to physiologically relevant concentrations of 4-MVA could further delineate its direct effects on inflammatory signaling pathways. Only if such experiments demonstrate reproducible, dose-dependent anti-inflammatory effects can 4-MVA be considered a bona fide effector molecule.

### Study limitations

Our findings not only reveal the important role of the “microbiota-metabolism-immunity” axis in the pathophysiology of endometriosis but also highlight the complexity of short-chain fatty acid metabolism regulation, particularly the identification of 4-MVA as a potential key effector molecule. However, this study has several limitations. First, the absence of a healthy control group (e.g., sham-operated rats) limits our ability to determine whether the observed probiotic effects represent a genuine restoration of healthy microbiota or merely a relative improvement within the disease state. Future studies should include such a control to further elucidate this issue. Second, a key limitation of our metabolite analysis is that only serum butyrate was measured, whereas serum levels of caproate and 4-MVA were not assessed. Consequently, our data cannot fully resolve whether the observed changes in fecal SCFA profiles reflect altered systemic availability of these metabolites. Future work should include comprehensive quantification of circulating SCFAs, including caproate and 4-MVA, to better link luminal changes to systemic exposure. Third, given the interspecies differences between rodent models and humans, the clinical implications of these mechanistic insights should be interpreted with caution. Further validation in models that more closely mimic human disease or in clinical samples is essential to substantiate these findings and inform the development of precise, microecology-based intervention strategies for EMs. Finally, the relatively small sample size of experimental rats may limit the statistical power of the results. We plan to expand the sample size in future work to strengthen the reliability of our conclusions.

## Conclusion

This study suggests that probiotic intervention may alleviate systemic inflammation in a rat model of endometriosis (EMs) by reshaping gut microbiota structure, enhancing microbial network stability, and regulating key metabolites, particularly short-chain fatty acids. 4-MVA is proposed as a candidate metabolite potentially involved in modulating EMs-related inflammation. However, as correlation with inflammatory markers did not reach statistical significance, the role of 4-MVA remains speculative and requires validation through dedicated mechanistic studies. Future investigations should employ larger sample sizes to further verify the specific functions of 4-MVA in EMs inflammatory regulation and to explore the underlying mechanisms of bile acid level changes.

## Data Availability

The raw sequencing data generated in this study have been deposited into a public repository. The data have been uploaded to the National Center for Biotechnology Information Sequence Read Archive and are accessible under the BioProject accession number PRJNA1378514. The original code and the input matrix are available at: https://gitlab.com/pengli1912-group/pengli1912-project.git.
